# Is heparinized 40% ethanol lock solution efficient for reducing bacterial and fungal biofilms in an *in vitro* model?

**DOI:** 10.1371/journal.pone.0219098

**Published:** 2019-07-08

**Authors:** Beatriz Alonso, María Jesús Pérez-Granda, María Consuelo Latorre, Carmen Rodríguez, Carlos Sánchez-Carrillo, Patricia Muñoz, María Guembe

**Affiliations:** 1 Instituto de Investigación Sanitaria Gregorio Marañón, Madrid, Spain; 2 Department of Clinical Microbiology and Infectious Diseases, Hospital General Universitario Gregorio Marañón, Madrid, Spain; 3 Cardiac Surgery Postoperative Care Unit, Hospital General Universitario Gregorio Marañón, Madrid, Spain; 4 CIBER Enfermedades Respiratorias-CIBERES (CB06/06/0058), Madrid, Spain; 5 Pharmacy Department, Hospital General Universitario Gregorio Marañón, Madrid, Spain; 6 Medicine Department, School of Medicine, Universidad Complutense de Madrid, Madrid, Spain; Karl-Franzens-Universitat Graz, AUSTRIA

## Abstract

**Background:**

We applied an *in vitro* model to evaluate the efficacy of a heparinized 40% ethanol-based lock solution in a wide variety of clinical isolates causing C-RBSI.

**Methods:**

A total of 100 clinical strains were collected retrospectively from the blood of patients with C-RBSI. The reduction in biomass and metabolic activity of biofilms was measured using the crystal violet (CV) assay and XTT assay, respectively. Regrowth inhibition (RI) was measured within 24 hours and 72 hours of ethanol lock therapy. Percentage reduction of ≥ 85% in RI was considered to be successful.

**Results:**

Ethanol lock was more effective in reducing metabolic activity than in reducing biomass (83% vs. 50%, respectively). Percentages of RI diminished as regrowth was prolonged (57% for 24 hours and 17% for 72 hours of regrowth). No statistically significant intraspecies differences were found in biofilm reduction or in RI (p>0.05).

**Conclusions:**

The use of heparinized 40% ethanol lock solution for 72 hours significantly reduced biomass and metabolic activity in clinical isolates from patients with C-RBSI. However, as biofilm has an important regrowth rate, 40% ethanol solution was not able to fully eradicate biofilm *in vitro*.

## Introduction

Catheter-related bloodstream infection (C-RBSI) is one of the most problematic nosocomial infections, with high rates of morbidity and mortality, high associated costs, and prolonged hospital stays in patients with long-term catheters that cannot be removed [1, 2]. The main causative agents of C-RBSI are gram-positive cocci, gram-negative bacilli, and yeasts, owing to their capacity to form robust biofilms on the catheter surface [3, 4].

When C-RBSI is suspected, guidelines recommend catheter withdrawal [5]. However, patients with long-term catheters and no alternative central access have to maintain their catheter in place [6]. In these cases, the main approach is to use conservative treatments based on the application of intravenous antibiotics combined with antibiotic lock therapy [1]. In a recent study published by Freire *et al*., ALT was successful in 75.9% of cancer patients, with an improvement in patient outcome [7]. However, increasing antimicrobial resistance rates are making antibiotic lock therapy less eligible; therefore, many other lock solutions are being tested [6, 8, 9].

Ethanol has high anti-biofilm activity. It is also easy to use and cost-effective, with no reports of associated resistance with promising results in clinical trials showing catheter salvage rates of between 71% and 100% [10–13]. Ethanol lock has been used widely for prophylactic proposes in C-RBSI [11, 12, 14, 15]. However the use of ethanol lock solutions for C-RBSI treatment is scarce [16]. Moreover, these solutions still have some controversies regarding dose, time of treatment, combination with anticoagulants and adverse effects [17, 18]. Hence, this study demonstrated that in an *in vitro* model ethanol at a relatively low concentration, such as 40% can be combined with heparin and can be effective in controlling C-RBSI. However, this study represents the first step in this kind of research as it. This solution must be evaluated in an *in vivo* model such as a murine model to analyse its efficacy and safety *in vivo* before being applied in clinical trials, which would be the last step of evaluation. Thus, our study opens new ways for ethanol lock solution research.

In a previous study by our group, a solution of 40% ethanol combined with 60 international units of heparin proved highly active against bacterial and fungal biofilms in ATCC strains [19]. However, the behavioral characteristics of ATCC strains differ from those of clinical strains [20]. Therefore, we applied an *in vitro* model to test the efficacy of a heparinized ethanol-based lock solution in a wide variety of clinical strains isolated from patients with C-RBSI. Hence, our study is the first to describe the efficacy of 40% ethanol-heparin lock solution in a large sample of clinical strains.

## Materials and methods

### Strains

A total of 100 clinical strains were collected retrospectively from the blood of patients with C-RBSI. Their distribution was as follows: 20 *Staphylococcus aureus* (10 methicillin-susceptible *S*. *aureus* and 10 methicillin-resistant *S*. *aureus*), 20 coagulase-negative staphylococci (CoNS) (10 *S*. *epidermidis*), 20 *Escherichia coli*, 20 *Enterococci* spp. (10 *E*. *faecium* and 10 *E*. *faecalis*), and 20 *Candida albicans*.

### Biofilm formation

Biofilms were grown as described in our previous study [19]. Briefly, a loopful of fresh culture was inoculated in 20 ml of medium and incubated at 30°C with shaking overnight. After 3 cycles of centrifugation-resuspension with PBS, inocula were adjusted to 0.5 or 0.35 McFarland for bacteria and yeast, respectively. One hundred microliters of each suspension were placed in a polypropylene 96-well microtiter plate and incubated at 37°C for 24 hours. Fresh medium (Sigma-Aldrich, Spain) was used as a negative control. Plates (Francisco Soria, Spain) were washed 3 times with PBS. All strains were tested in triplicate.

### Ethanol lock treatment

Each well was treated with 120 μl of 40% ethanol and 60 international units of heparin (heparin sodium, 5,000 IU/5 ml, Hospira Prod. Farm. y Hosp, S.L.) plus 120 μl of medium at 37°C for 72 hours. Positive controls were treated only with 120 μl of fresh medium. Ethanol solution and medium were replaced every 24 hours.

### Biomass reduction assay

Reduction in biofilm biomass was evaluated using crystal violet (CV) dye. After ethanol treatment, plates were washed 3 times with PBS. After plates were completely dry, 200 μl of 99% methanol were added for 10 minutes at room temperature. Methanol was discarded, and 125 μl of 0.1% CV was added for 10 minutes at room temperature. Plates were washed with distilled water, and fixed CV was released using 125 μl of 30% acetic acid for 15 minutes. The volume was transferred to a new plate, and absorbance (550 nm) was measured in a spectrophotometer (Biochrom EZ Read 400, Mervilab, Spain). The percentage of reduction was calculated using **Eq 1**.

$\%reduction\;in\;biomass=\left(1-\frac{{Abs}_{550\;treated\;sample}}{{Abs}_{550\;positive\;control}}\right)*100$**Eq 1.** Percentage of biomass reduction after ethanol lock therapy.

### Metabolic activity reduction assay

Plates were washed 3 times with PBS, and metabolic activity was measured by adding 100 μl of a premixed solution of 10 ml of XTT (0.5 mg/ml) (Sigma-Aldrich, Spain) with 40 μl of menadione (1.72 mg/ml) (Sigma-Aldrich, Spain) protected from light. Plates were incubated at 37°C for 2 hours, and absorbance was measured at 492 nm in a spectrophotometer (Biochrom EZ Read 400, Mervilab, Spain). The percentage of reduction in metabolic activity was calculated using **Eq 2**.

$\%reduction\;in\;metabolic\;activity=\left(1-\frac{{Abs}_{492\;treated\;sample}}{{Abs}_{492\;positive\;control}}\right)*100$**Eq 2.** Percentage of reduction in metabolic activity after ethanol lock therapy.

### Regrowth inhibition

After ethanol therapy was administered and wells were washed 3 times with PBS, 100 μl of fresh medium was added and incubated for 24 hours and 72 hours at 37°C. The medium was replaced every 24 hours. Absorbance was measured at 492 nm, and the percentage of regrowth inhibition (RI) for each incubation period was calculated using **Eq 3.**

$\%RI=\left(\frac{{Abs}_{492\;positive\;control}-{Abs}_{492\;treated\;sample}}{{Abs}_{492\;positive\;control}}\right)*100$**Eq 3.** Percentage of regrowth inhibition within 24 and 72 hours after ethanol lock therapy.

### Statistical analyses

Data were expressed as mean (SD) for percentage reduction in biomass, metabolic activity, and RI. We considered a percentage of ≥85% as successful RI according to clinical data on the success of antibiotic lock therapy [21].

Quantitative variables were compared using an ANOVA test and Games-Howell post hoc test. Qualitative variables were compared using the chi-square test. Statistical significance was set at p<0.05. All tests were performed using the statistical program SPSS 21.0 (IBM).

## Results

Ethanol lock solution was successful in decreasing biomass and metabolic activity in 50% and 83% of cases, respectively. **Table 1** describes the distribution of all bacteria and fungal species in which treatment was successful. Ethanol showed better anti-biofilm activity for XTT than for CV in all species.

**Table 1:** Distribution of strains in which 40% ethanol-60 IU heparin lock solution for 72 hours proved successful.

Microorganism	Success rate*, N (%)	
	CV	XTT
*S*. *aureus* (N = 20)	7 (35)	8 (40)
CoNS (N = 20)	5 (25)	16 (80)
*Enterococci sp*. (N = 20)	19 (95)	19 (95)
*E*. *coli* (N = 20)	19 (95)	20 (100)
*C*. *albicans* (N = 20)	0 (0)	20 (100)
**Total (N = 100)**	**50 (50)**	**83 (83)**

**IU;** international units; **CV**, crystal violet; **XTT,** 2,3-Bis-(2- methoxy 4-nitro-5-sulfophenyl)-2H-tetrazolium5-carboxanilide salt; **CoNS**, coagulase-negative staphylococci.

*Success rate was set as ≥85%.

**Fig 1:**
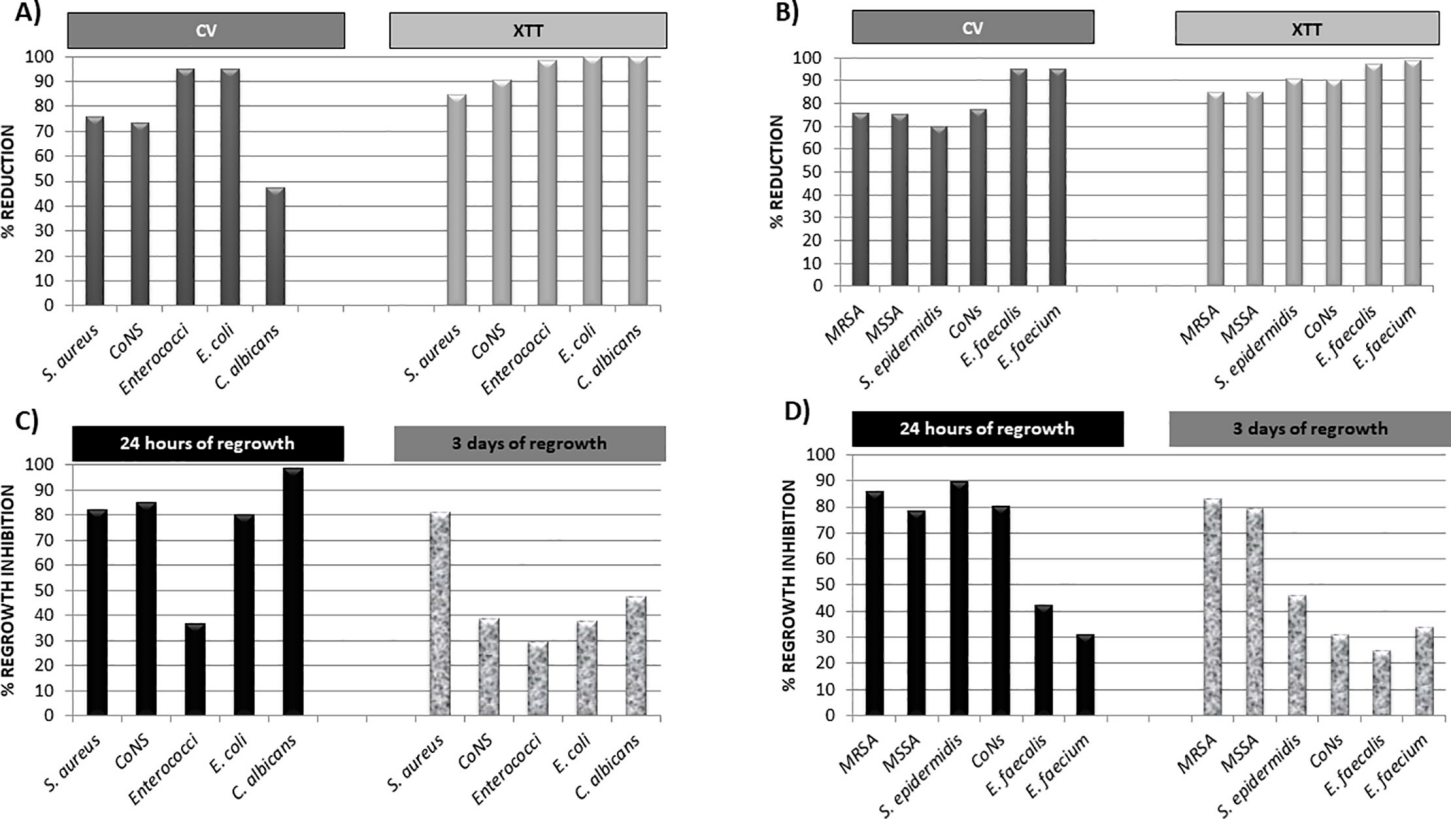
Percentage reduction of biomass and metabolic activity and percentage of regrowth inhibition of biofilms after 72-hour treatment of 40% ethanol combined with 60 international units (IU) of heparin. **A)** Overall percentage reduction in biomass and metabolic activity after 72-hour treatment with heparinized 40% ethanol lock solution. **B)** Percentage reduction in biomass and metabolic activity after 72-hour treatment with heparinized 40% ethanol lock solution according to species. **C)** Overall percentage of regrowth inhibition within 24 hours and 72 hours after 72-hour treatment with heparinized 40% ethanol lock solution. **D)** Percentage of regrowth inhibition within 24 hours and 72 hours after 72-hour treatment with heparinized 40% ethanol lock solution according to species. **CV**, crystal violet; **XTT,** 2,3-Bis-(2- methoxy 4-nitro-5-sulfophenyl)-2H-tetrazolium5-carboxanilide salt; **CoNS**, coagulase-negative staphylococci; **MRSA,** methicillin-resistant *Staphylococcus aureus*; **MSSA,** methicillin-susceptible *Staphylococcus aureus*.

The overall percentages of reduction for CV and XTT assays are shown in **Fig 1A**. Percentages ranged between 47.5% (*C*. *albicans*) and 95.2% (*Enterococci sp*.) (p<0.001) for biomass reduction and 84.8% (*S*. *aureus*) and 100% (*E*. *coli*) (p<0.001) for metabolic activity reduction. No statistically significant intraspecies differences were found (p>0.05) (**Fig 1B**).

As for RI after ethanol lock, rates decreased for all strains from 57% to 17% of success within 24 hours and 72 hours, respectively (**Table 2**). After 24 hours of regrowth, inhibition ranged from 98.5% (*C*. *albicans*) to 36.8% (*Enterococci sp*.) (p<0.001). In contrast, when regrowth was assessed within 72 hours, percentages of inhibition varied between 81.2% (*S*. *aureus*) and 29.4% (*Enterococci sp*.) (p<0.001). RI for *S*. *aureus* was ≥80% in both periods.

**Table 2:** Percentages of success in regrowth inhibition (RI) after 40% ethanol-60 IU heparin lock solution for 72 hours.

Microorganism	Success rate* of RI, N (%)	
	24 hours	72 hours
*S*. *aureus* (N = 20)	13 (65)	10 (50)
CoNS (N = 20)	12 (60)	1 (5)
*Enterococci sp*. (N = 20)	1 (5)	0 (0)
*E*. *coli* (N = 20)	11 (55)	4 (20)
*C*. *albicans* (N = 20)	20 (100)	2 (10)
**Total (N = 100)**	**57 (57)**	**17 (17)**

**CoNS**, coagulase-negative staphylococci.

*Success rate for RI was set as ≥85%.

**Fig 1C** shows the overall results for RI. No statistical intraspecies differences were found at 24 hours or at 72 hours after ethanol lock therapy (p>0.05) (**Fig 1D**).

## Discussion

We found that 40% ethanol plus 60 IU of heparin was able to reduce metabolic activity by up to 85% in 5 of the most causative agents of C-RBSI after 72 hours of locking. However, these strains were able to regrow within 72 hours after ethanol therapy.

Although the frequency of C-RBSI has decreased in the last decade, this condition still represents a huge challenge in clinical settings, with high associated costs (€18,000€/episode), high mortality (up to 25%), and longer hospitalizations [22, 23]. Thus, research has focused on prophylaxis and treatment of C-RBSI in patients with no possibility of catheter replacement using different agents as lock therapy [24]. Antibiotics are the most common agent for lock therapy [25]. However, overuse of antibiotics is increasing the frequency of multidrug-resistant strains [6]. Ethanol has been proposed as an alternative to antibiotics in lock therapy [12, 13]. However, most clinical studies used 70% ethanol, which shows important adverse effects such as ethanol taste, nausea, dizziness, rupture of catheter lumen, or catheter occlusion [17]. In our previous study, we demonstrated that 40% ethanol for 72 hours was sufficient to reduce the metabolic activity of biofilm in ATCC strains [19]. Furthermore, this concentration of alcohol can be safely combined with heparin, which is required for locks of 24 hours or more [19]. In our study we demonstrated that this ethanol solution is also efficient in reducing biofilms of C-RBSI clinical isolates.

Our results are consistent with those of other *in vitro* studies. Öncü *et al*. reported the anti-biofilm activity of 40% ethanol in *Candida* species [26]. Using 1 cm of silicon catheter, the authors demonstrated that 40% ethanol was able to inhibit growth of clinical *C*. *albicans* isolates in Sabouraud dextrose agar within 30 minutes [26]. Using the XTT assay, Peters *et al*., [27] reported complete inhibition of metabolic activity with 40% ethanol for 4 hours in *C*. *albicans* and *S*. *aureus* prototype strains. Moreover, Chaudhury *et al*. and Qu *et al*., did not recover any colonies of *S*. *aureus* or CoNS after 1 hour of 40% ethanol lock therapy [28, 29]. In the case of gram-negative bacilli, Chamber *et al*., showed bactericidal effects when 30%-90% ethanol was applied to mature biofilms for 8 hours [30].

While very impressive in terms of reduced metabolic activity, our results show that regrowth inhibition was low in almost all the tested species within 24 hours and 72 hours after ethanol lock therapy. Regrowth of almost all the strains did not achieve a 100% reduction, except for *E*. *coli*, which showed a 95.1% reduction in biomass, suggesting that some metabolically active cells were still able to resuscitate after the addition of fresh medium. This phenomenon, known as viable but non-culturable (VBNC) cells, remains markedly challenging for clinicians and accounts for the high rates of treatment failure [31]. We hypothesize that this kind of cell could be present in all tested strains, as the factors inducing this cellular state include environmental stress and changes in pH, both of which are associated with exposure to ethanol [32–34]. Moreover, percentages of reduction in biomass and metabolic activity differed substantially, thus supporting the conversion of active cells to VBNC cells. Although the CV and XTT assays are complementary for biofilm characterization [35], CV can dye viable cells that cannot be detected using XTT, as they are metabolically inactive. Furthermore, Parasuraman *et al*. have demonstrated in a new study that confocal microscopy could be very useful in evaluating the cell viability which would be very useful for detecting VBNC cells and hence, the real efficacy of ethanol lock solution [36]. The authors also demonstrated the usefulness of nanoparticles for inhibiting biofilm formation and extracellular matrix production [36]. The use of ethanol encapsulated in nanoparticles could improve ethanol penetration in the inner layers of biofilms and thus, eradicating the biofilm and completely inhibit cell regrowth.

To the best of our knowledge, this is the first *in vitro* study to describe the anti-biofilm activity of ethanol in such a large collection of clinical C-RBSI strains, with the novelty of adding heparin in the lock solution. Furthermore, given that ethanol lock therapy has not previously been reported to be efficacious for *Enterococci sp*., our findings provide new insight into the treatment of C-RBSI.

Our study is subject to limitations. First, the *in vitro* model does not mimic clinical conditions and is subject to high biological variations even between wells [37]. However, this system is ideal for screening purposes and for testing different antibiotics and disinfectants in a large numbers of strains simultaneously [37]. Second, we did not count colony-forming units, although our results are similar to those obtained by other authors [26, 28, 29]. Third, we only studied the efficacy of ethanol lock therapy in monomicrobial biofilms and it would be necessary to assess this efficacy in polymicrobial biofilms due to the clinical relevance that they represent.

In conclusion, our results provide a good preliminary insight into the effect of 40% ethanol-60 IU heparin as lock solution for C-RBSI. Although future studies are needed to improve ethanol penetration and its action in VBNC cells, this lock solution could be an alternative to conservative antibiotic lock therapy solutions for maintaining catheters until they can be replaced.

## Supporting information

S1 Text Syntaxis text for statistical analysis. (SPS)

S1 Table Database of the strains used in the study. (XLSX)

S2 Table Database of absorbance values for crystal violet, XTT and regrowth inhibition assays. (SAV)

S3 Table ANOVA test results for regrowth inhibition analysis. (ZIP)
