# Correction: Is heparinized 40% ethanol lock solution efficient for reducing bacterial and fungal biofilms in an *in vitro* model?

**DOI:** 10.1371/journal.pone.0221702

**Published:** 2019-08-22

**Authors:** Beatriz Alonso, María Jesús Pérez-Granda, María Consuelo Latorre, Carmen Rodríguez, Carlos Sánchez-Carrillo, Patricia Muñoz, María Guembe

The Health Research Fund (FIS) grant number is missing. The correct grant number is PI18/00045.
